# Progressive Multifocal Leukoencephalopathy or Lymphoma? A Massive Unilateral Hemispheric Mimicker in a Patient Undergoing Lymphoma Treatment

**DOI:** 10.3390/reports9020170

**Published:** 2026-05-29

**Authors:** Koji Hayashi, Mamiko Sato, Hiroki Tsukamoto, Eiju Negoro, Takahiro Yamauchi

**Affiliations:** 1 Department of Rehabilitation Medicine, Fukui General Hospital, 55-16-1 Egami-cho, Fukui 910-8561, Japan; satomoko@f-gh.jp; 2 Graduate School of Health Science, Fukui Health Science University, 55-13-1 Egami-cho, Fukui 910-3190, Japan; 3 Department of Hematology and Oncology, University of Fukui Hospital, 23-3 Matsuoka Shimoaizuki, Eiheiji-cho, Yoshida-gun, Fukui 910-1193, Japan; htsukamo@u-fukui.ac.jp (H.T.); enegoro@u-fukui.ac.jp (E.N.); tyamauch@u-fukui.ac.jp (T.Y.)

**Keywords:** leukoencephalopathy, progressive multifocal, JC virus, lymphoma, follicular, magnetic resonance imaging, diagnosis, differential, case report

## Abstract

An 81-year-old woman with follicular lymphoma treated with obinutuzumab and bendamustine developed cognitive impairment and dysarthria. Three months before death, neurological exams showed dysarthria, right hemiparesis, and gait disturbance. Blood tests showed lymphocytopenia (lymphocyte 10.4%). Cerebrospinal fluid (CSF) findings were unremarkable, including with respect to cytology. Brain MRI demonstrated a mass-like hyperintense lesion in the left parietal lobe and band-like abnormalities in the left fronto-temporal white matter that lacked contrast enhancement. Symptoms progressed to hemiplegia and mutism; severe dysphagia eventually necessitated intravenous fluid management. Follow-up MRI one month before death revealed a lesion encompassing nearly the entire left hemisphere, with hyperperfusion observed during arterial spin labeling (ASL). JC virus was detected in CSF (221 copy/mL), confirming that the patient had progressive multifocal leukoencephalopathy (PML). Subsequently, she exhibited poor arousal, followed by death. Here, lymphoma recurrence or PML was suspected due to a post-chemotherapy unilateral expanding brain lesion. These conditions are usually differentiated by contrast-enhancement patterns, but PML can also enhance during immune reconstitution. Moreover, lesions rarely cause a mass effect and more often exhibit hyperperfusion, which may aid in diagnosis. While unilateral PML has been reported, especially in the early stage, such an extensive lesion involving nearly an entire single hemisphere, as seen in our case, is rare.

**Figure 1:**
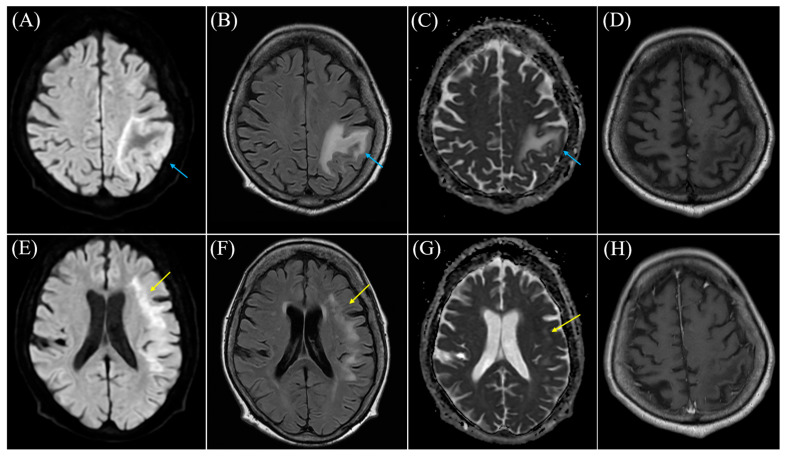
The brain magnetic resonance imaging (MRI) results obtained 3 months prior to death. (**A**,**E**) Diffusion-weighted imaging (DWI). (**B**,**F**) T2-Fluid attenuated inversion recovery (T2-FLAIR) images. (**C**,**G**) Apparent diffusion coefficient (ADC) maps. (**D**) T1-weighted imaging. (**H**) Contrast-enhanced T1-weigted imaging. DWI and T2-FLAIR images revealed a mass-like hyperintense lesion in the left parietal lobe (blue arrow), along with band-like hyperintensities extending from the frontal to the temporal lobes and involving subcortical U-fibers and deep white matter (yellow arrow). The corresponding ADC maps of these DWI hyperintense regions showed high values. The patient was undergoing treatment for malignant lymphoma, and the parietal-lobe MRI abnormalities appeared mass-like, necessitating exclusion of lymphoma metastasis or intravascular lymphoma. Cerebrospinal fluid (CSF) analysis revealed no pleocytosis or elevated protein levels, and cytology showed no abnormalities. Furthermore, there was no enhancement on contrast-enhanced MRI, supporting a low likelihood of malignant lymphoma. While a brain biopsy would have definitively excluded lymphoma, it was deferred as the patient met the criteria for ‘definite progressive multifocal leukoencephalopathy (PML)’ per the American Academy of Neurology consensus statement [1]. This diagnosis was firmly supported by the combination of progressive clinical deficits, the detection of JC virus DNA in the CSF (221 copies/mL), and the hallmark radiological finding of a massive lesion with a conspicuous absence of mass effect. Both obinutuzumab and bendamustine have been reported to be associated with a risk of PML [2,3]. Nonetheless, it is notable that PML typically shows no contrast enhancement on MRI in classic cases, but contrast enhancement often appears during immune reconstitution inflammatory syndrome (IRIS), reflecting inflammatory response [4,5,6,7,8].

**Figure 2:**
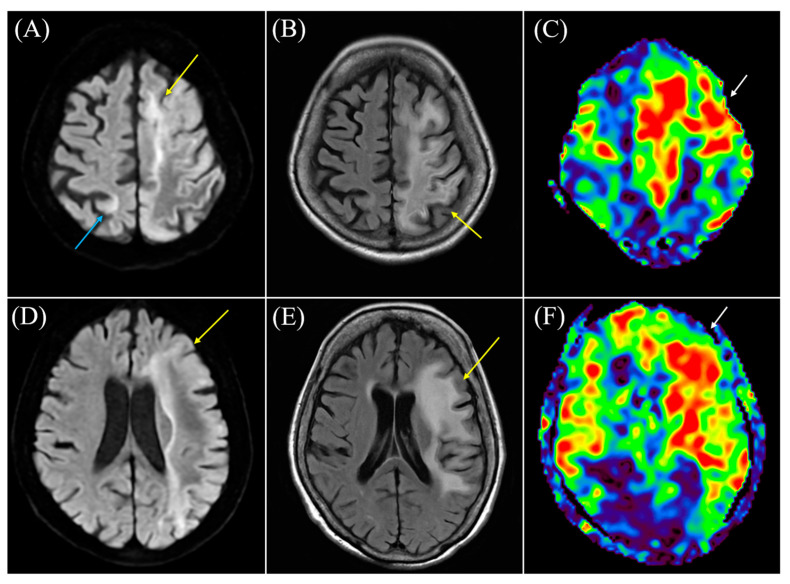
Follow-up brain MRI obtained one month prior to death. (**A**,**D**) Relative to the previous brain MRI, the DWI abnormalities have spread throughout the left cerebral hemisphere, extending into parts of the white matter (yellow arrow). A small area with a high DWI signal was also seen in the right parietal lobe (blue arrow). (**B**,**E**) The DWI abnormal signal areas are slightly more extensive than on T2-FLAIR (yellow arrow). (**C**,**F**) Hyperperfusion was noted in a slightly widespread area centered around the lesion (white arrow). Blue: low perfusion; red: high perfusion. (**A**,**D**) DWI imaging. (**B**,**E**) T2-FLAIR imaging. (**C**,**F**) Arterial spin labeling (ASL) imaging. PML typically manifests as a bilateral, asymmetric, and multifocal white-matter disease; however, it can occasionally present as a unilateral condition with a solitary lesion, especially in the early stage [9]. Early detection through MRI screening has revealed that unilateral lesions are significantly more common in asymptomatic patients (68%) than in symptomatic individuals (37%) [10]. While focal PML lesions generally enlarge and become confluent within the white matter—eventually spreading to non-contiguous lobes to establish a widespread bilateral distribution [10]—unilateral or single-lesion presentations are sometimes associated with “atypical” PML occurring in immunocompetent hosts or patients with minimal overt immunosuppression [9,11]. Clinically, unilateral PML often presents as slowly progressive hemiplegia, sensory loss, or visual-field deficits such as hemianopsia [11,12,13]. Notably, patients diagnosed at this localized stage have a substantially higher survival rate (85%) compared to those with widespread disease (69%) [13]. However, the focal nature of unilateral PML presents significant diagnostic challenges, as it is frequently misdiagnosed as subacute cerebral infarction, tumefactive multiple sclerosis, or a brain tumor [9,11,13], although the neuroradiology pattern can suggest the right diagnosis. In our case, the patient was undergoing treatment for follicular lymphoma, and the initial MRI abnormality in the left parietal lobe appeared strikingly mass-like, necessitating the exclusion of CNS lymphoma recurrence or metastasis. However, a key diagnostic differentiator is that PML typically produces minimal to no mass effects, unlike the significant displacement of brain structures often seen with malignant tumors [9,10]. Remarkably, even as the lesion in our patient expanded to involve nearly the entire left hemisphere, there was no significant midline shift or compression of the ventricular system, reinforcing the “clinical paradox” characteristic of PML. This clinical paradox—a massive “mass-like” lesion without an actual mass effect—served as a crucial radiological clue with which to differentiate this extensive PML from a neoplastic process. In PML, MRI lesions typically manifest as T2/FLAIR hyperintense, multifocal, bilateral, and asymmetric alterations that are usually located in the cerebral white matter (most often subcortically), cerebellum, and brainstem [14]. Crescent-shaped cerebellar lesions are considered characteristic radiological findings [14,15]. These lesions typically lack a significant mass effect or perilesional edema, and recognizing a pattern of restricted patchy peripheral diffusion may further aid in the diagnosis [14]. Regarding contrast-enhanced MRI, enhancement is usually absent or modest [14,15]. Furthermore, the diagnostic reliability of contrast-enhanced MRI is limited by the temporal variability of PML lesions, but this was not a relevant question in our case, as no enhancement was observed. While contrast enhancement is typically absent in classic cases, it can emerge in 30–40% of early-stage presentations (frequently seen in natalizumab-associated cases) or during the development of IRIS [10]. Such stage-dependent enhancement patterns—often appearing as punctate or patchy signals at the lesion periphery [10]—frequently overlap with the characteristics of other pathologies, significantly complicating the differential diagnosis with respect to malignancies like lymphoma. Advanced imaging, specifically ASL, provides critical diagnostic clues in this case. Hyperperfusion observed during ASL imaging has been reported as a characteristic finding in active PML lesions and is often associated with disease progression in the absence of IRIS [12,16,17,18,19]. The hyperperfusion in our case was localized and extended slightly beyond the structural lesion, differing from the global hyperperfusion typically seen in IRIS. Given the complete absence of contrast enhancement—a hallmark of IRIS—this focal hyperperfusion might represent the ‘advancing front’ of active viral replication and associated inflammation. These findings suggest that ASL can serve as a valuable non-invasive biomarker for identifying early-stage PML, particularly when traditional contrast-enhanced MRI is inconclusive, thereby facilitating a more prompt and accurate diagnosis for immunocompromised patients. This case provides a clinically critical lesion regarding the “radiological paradox” essential for differentiating PML from malignancy. While the massive, unilateral lesion initially mimicked a neoplastic process, the conspicuous absence of a mass effect—despite its extensive size—provided the defining diagnostic clue. Although unilateral or localized PML is typically associated with earlier stages or a more favorable prognosis, our case demonstrates that such laterality can be strikingly persistent and aggressive in an immunocompromised host, leading to a fatal outcome. To our knowledge, this is the first report of such extensive, hemispheric PML involvement persistently localized to a single side in a patient under potent immunosuppression. The patient was treated with obinutuzumab, which induces B-cell depletion, and bendamustine, known for its T-cell suppressive effects. While the synergistic action of these two agents may have created a unique immune environment in this patient, the precise mechanism explaining why the lesion remained almost strictly unilateral despite its massive extent remains to be elucidated. Whether this specific chemoimmunotherapy regimen consistently correlates with such a unique ‘hemispheric’ phenotype of PML is a critical question. Validating this hypothesis will require further clinical case accumulation and integrated immunological studies, which are essential for improving the diagnostic accuracy of PML in regard to patients undergoing these potent lymphoproliferative treatments. Clinicians must remain vigilant; understanding this paradoxical presentation is vital to prompting early JC virus testing and intercepting the disease before it encompasses an entire hemisphere.

## Data Availability

The data presented in this study are available on request from the corresponding author. Due to patient privacy and ethical considerations, the data are not publicly accessible.
